# Raman micro-spectroscopy of two types of acetylated Norway spruce wood at controlled relative humidity

**DOI:** 10.3389/fpls.2022.986578

**Published:** 2022-09-06

**Authors:** Andrea Ponzecchi, Emil E. Thybring, Ramūnas Digaitis, Maria Fredriksson, Sara Piqueras Solsona, Lisbeth Garbrecht Thygesen

**Affiliations:** ^1^Bioresource Chemistry and Technology, Department of Geoscience and Natural Resource Management, University of Copenhagen, Frederiksberg, Denmark; ^2^Division of Building Materials, Lund University, Lund, Sweden; ^3^Department of Wood Technology, Norwegian Institute of Bioeconomy Research, Ås, Norway; ^4^Department of Wood and Biomaterials, Danish Technological Institute, Taastrup, Denmark

**Keywords:** Raman micro-spectroscopy, wood, acetylation, biological imaging, chemical modification, moisture, relative humidity, water

## Abstract

Water is a key element for wood performance, as water molecules interact with the wood structure and affect important material characteristics such as mechanical properties and durability. Understanding wood-water interactions is consequently essential for all applications of wood, including the design of wood materials with improved durability by chemical modification. In this work, we used Raman micro-spectroscopy in combination with a specially designed moisture chamber to map molecular groups in wood cell walls under controlled moisture conditions in the hygroscopic range. We analyzed both untreated and chemically modified (acetylated to achieve two different spatial distributions of acetyl groups within the cell wall) Norway spruce wood. By moisture conditioning the specimens successively to 5, 50, and 95% relative humidity using deuterium oxide (D_2_O), we localized the moisture in the cell walls as well as distinguished between hydroxyl groups accessible and inaccessible to water. The combination of Raman micro-spectroscopy with a moisturizing system with deuterium oxide allowed unprecedented mapping of wood-water interactions. The results confirm lower moisture uptake in acetylated samples, and furthermore showed that the location of moisture within the cell wall of acetylated wood is linked to the regions where acetylation is less pronounced. The study demonstrates the local effect that targeted acetylation has on moisture uptake in wood cell walls, and introduces a novel experimental set-up for simultaneously exploring sub-micron level wood chemistry and moisture in wood under hygroscopic conditions.

## Introduction

Durability is a factor that often limits the service life of wood and wood products, especially for in-soil and outdoor applications. The use of wood in outdoor environments is challenging as the wood cell wall will be degraded by decay fungi when exposed to prolonged humid conditions. Moisture plays a key role in this process, as it is essential for fungi to colonize and consume the lignocellulosic cell walls (Ringman et al., 2019; Brischke and Alfredsen, 2020). As a hygroscopic material, wood takes up water from its surroundings both in vapor and liquid state. Hydroxyl (OH) groups are the main water sorption sites in wood cell walls (Simpson, 1980) and these are present throughout the lignocellulosic matrix. Chemical modification of wood is a way to improve the durability, often by limiting the hygroscopicity of the material (Dong et al., 2020). Modifications of wood can limit the access of water molecules in the cell wall by bulking the available space and/or by reducing the number of accessible sorption sites in the structure (Thybring and Fredriksson, 2021). The most utilized wood modification processes are acetylation, thermal modification and furfurylation (Rowell, 2006; Mantanis, 2017; Hill et al., 2021; Zelinka et al., 2022). Acetylation of wood by reaction with acetic anhydride substitutes a fraction of the hydroxyl groups with the more voluminous acetyl groups (Çelen et al., 2007). Since the cell wall chemistry of wood is heterogeneous, chemical modification may not affect all domains evenly. The spatial distribution of a chemical modification can also be deliberately controlled by tuning the reaction conditions (Digaitis et al., 2021) or the reaction path (Keplinger et al., 2015). Chemical changes in wood cell walls as a result of modification are often studied by Raman micro-spectroscopy because it is non-invasive and offers high spatial resolution (Agarwal, 2009, 2019; Gierlinger et al., 2012, 2013; Gierlinger, 2018). The chemical characterization has so far been conducted predominantly on water-saturated wood specimens and information related to non-saturated states is limited (Guo et al., 2017). In this study we introduce a novel combination of Raman micro-spectroscopy and controlled moisture conditioning of wood in a unique, custom-built moisture chamber. With this experimental setup we are able to study water within wood cell walls under controlled, non-saturated environmental conditions. Moreover, by use of deuterium exchange, water-accessible and non-accessible hydroxyl groups can be distinguished from each other. This allows visualization of the moisture distribution within cell walls of native and modified wood. Here, we demonstrate this setup and semi-quantitatively assess the distribution of acetyl groups and moisture within native and two types of acetylated wood cell walls of Norway spruce to illustrate local effects of acetylation.

## Materials and methods

### Wood material

Wood specimens of untreated, pyridine treated (controls), uniformly acetylated and interface acetylated Norway spruce [*Picea abies* (L.) Karst.] mature sapwood with dimensions 10 (longitudinal) × 5 × 5 mm^3^ were employed for this study. The material originated from experimental forests in the southern parts of Sweden and is further described by Fredriksson et al. (2016). To minimize variation between specimens all specimens were cut from the same board. The modification procedures are described in detail by Digaitis et al. (2021). Briefly, the uniform acetylation was achieved by impregnating the samples in a 1:4 (v/v) mixture of acetic anhydride (VWR Chemicals, Radnor, United States) and pyridine (Merck, Darmstadt, Germany) and subsequent heating at 80°C for 60 min. The interface acetylation was achieved using a solution of pure acetic anhydride and carrying out the reaction at 75°C for 24 h. Control specimens were treated with pure pyridine at 80°C for 3 h. The mass gain caused by the modification was evaluated as the relative mass change:


(1) $$R_{\mathrm{mod}}=\frac{m_{\text{dry}}-m_{\text{dry,0}}}{m_{\text{dry,0}}}$$

where *m*_*dry*_ (g) is the dry mass after modification and *m*_*dry,0*_ (g) is the dry mass before modification.

The recorded *R*_mod_ (g/g) for the interface acetylated specimen used in this study was 0.113 g/g. The mean *R*_mod_ of 10 uniformly acetylated specimens was 0.142 g/g. The pyridine extraction gave on average a negative *R*_mod_ of 0.023 g/g, indicating a mass loss, possibly due to removal of extractives from the wood.

### Raman measurements with controlled humidity

Using a microtome (RM2255, Leica Biosystems, Wetzlar, Germany), three 16 μm cross-sections were produced per specimen, in total 3 × 4 = 12 cross-sections. Four cross-sections at the time, one per each type of wood material, were placed on the moisture chamber (detailed description of the moisture chamber used is provided in Supplementary Material, Section 1) and wetted with a drop of deuterium oxide (99.98% D_2_O, Sigma-Aldrich, Munich, Germany). The samples were then covered with a borosilicate glass slide (thickness #1), the edges of which were sealed with nail polish. The fully assembled and loaded with wood cross-sections moisture chamber, with open inlet and outlet channels, was then vacuum dried for 12 h at 60°C. Afterward, the moisture chamber was connected to a humidity microcontroller (ACE flow 2.0, SolGelWay, France) to adjust the flow of a wet flux of saturated deuterium oxide (D_2_O) vapor and a dry flux of air at 0% RH. The water saturated flux was achieved with a bubbling system that included two flasks, a warming plate and D_2_O (Supplementary Figure 1). To ensure that all water-accessible hydroxyl groups were deuterated, the samples were preconditioned at 95% RH for 12 h. Then, the samples were equilibrated at 5, 50, and 95% RH for 12 h at each humidity level, and at each of these humidity levels, Raman images were captured. A total of 36 Raman images were captured, describing 12 different latewood tracheids at three hygroscopic states, belonging to four types of wood material.

The confocal Raman microscope (alpha 300R, WITec GmbH, Ulm, Germany) was equipped with a UHTS 300 spectrometer and a 100x oil immersion objective (Zeiss “N-Achroplan,” NA = 1.2, transmittance of 73%, Carl Zeiss GmbH, Jena, Germany). A linear polarized 532 nm NdYag was used at a 10 mW laser power and with 0.1 s of integration time per spectrum to avoid sample degradation (Prats-Mateu et al., 2018). Even though the same tracheid was imaged three times, no signs of degradation were observed in the spectra. The images were acquired from cross sections that were previously aligned with the tangential direction parallel to the laser polarization (Gierlinger et al., 2013). Raman scattered light was detected with a back-illuminated charge-coupled device camera, air cooled with Peltier cooling to –60°C and with a 600 g/mm grating, resulting in a spectral resolution of 3.8 cm^–1^. Images were acquired with a diffraction limited lateral spatial resolution of approximately 0.3 μm.

### Treatment and data analysis of Raman scattering data

The treatment and reduction of all Raman scattering data were carried out in Matlab ver. 2020b (Mathworks, Natick, Massachusetts, United States). Prior to analysis, spectra were subjected to (1) image size reduction, specifically shaped for each image, to reduce the size of the dataset; (2) removal of the part of the spectrum not useful for the analysis, consisting in the wavenumbers below 300 cm^–1^ and above 3,720 cm^–1^ approximately; (3) cosmic ray removal by use of median filtering (Matlab built-in function medfilt1 using default settings); (4) Alternating Least Squares (ALS) baseline correction according to Eilers and Boelens (2005), which has been shown to cope well with fluorescence contribution (De Juan et al., 2014), with parameters λ = 10^5^ and *p* = 0.0005. Due to the heterogeneous distribution of wood polymers in the wood cell walls, the data were clustered using k-means cluster analysis (as implemented in Matlab), which successfully separated lignin rich parts of the cell wall, i.e., the cell corner and middle lamella (CCML), the cellulose rich secondary cell wall (S2), and the empty lumina of tracheids and ray cells (LUMEN). A normalization to equal length (2-norm of each spectrum) was used before clustering, as it made the k-means clustering perform better based on our visual inspection of the clustering results.

**FIGURE 1:**
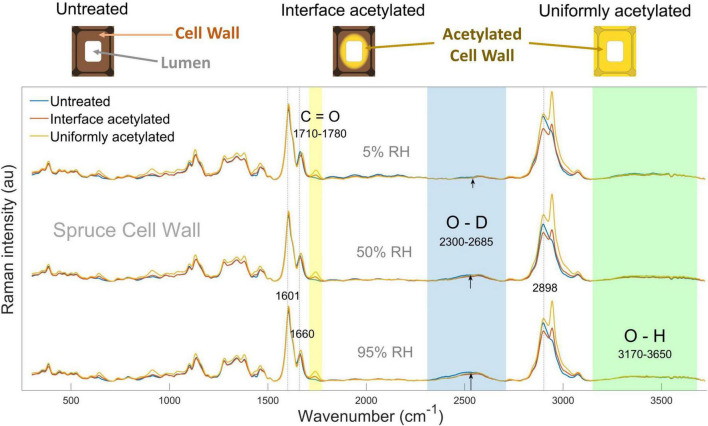
In the top, schematic representations of untreated, interface and uniformly acetylated cell walls are shown. Below, average Raman spectra calculated for untreated (blue), interface acetylated (red), and uniformly acetylated (yellow) spruce cell walls (i.e., CELL WALL cluster), conditioned in D_2_O vapor at 5% (top), 50% (middle), and 95% RH (bottom). Wavenumber regions of interest assigned to acetylation, moisture, and water-inaccessible hydroxyls are shaded, respectively, in yellow, blue, and green. Peaks of interests are also highlighted by dotted lines, and marked with their exact wavenumber. The spectra for pyridine control samples are omitted for clarity as they were similar to the untreated wood, see Supplementary Figure 2.

For the spectra assigned to the cell wall cluster (CELL WALL = S2 *+* CCML clusters), average spectra were computed and Raman peak heights or areas were estimated using a linear baseline, individually set for each Raman band. Estimation of peak areas was preferred over peak heights when possible, i.e., when the peak of interest was an isolated peak and not a shoulder. For the sake of visual comprehension, in addition to the pre-processing, the average spectra of the cell walls in Figure 1 have been furthered baseline corrected (ALS, λ = 10^4^ and *p* = 0.0002). Peak areas were estimated with trapezoidal numerical integration (Matlab trapz function), and peak heights by the height of the baselined corrected peak, using a linear baseline individually set for each Raman band of interest. Peak areas were estimated for: (1) O-D stretching at 2,490 cm^–1^ (Hofstetter et al., 2006), calculated in the range 2,300–2,685 cm^–1^ and assigned to the absorbed deuterium oxide (D_2_O) and the deuterated hydroxyls (O-D); (2) C=O carbonyl stretch at 1,738 cm^–1^ (Marchessault and Liang, 1962; Adebajo et al., 2006), in the range between 1,710 and 1,780 cm^–1^ and assigned to acetylation and (3)

O-H stretching at 3,450 cm^–1^(Wiley and Atalla, 1987), in the range between 3,150–3,650 cm^–1^ and assigned to inaccessible hydroxyl groups. Peak heights were estimated for: (1) the mean aromatic ring stretching at 1,601–1,604 cm^–1^ (Gierlinger and Schwanninger, 2007), using a baseline in the range between 1,545 and 1,710 cm^–1^ and assigned to the symmetric CC stretch of the aromatic ring of lignin substructures (Bock and Gierlinger, 2019); (2) the mean of the C-H stretch at 2,898–2,902 cm^–1^ (Gierlinger et al., 2013) using a baseline in the range 2,785–3,040 cm^–1^; (3) the maximum height of the C=C and C=O stretch calculated between 1,660 and 1,664 cm^–1^(Bock and Gierlinger, 2019), using a baseline in the range between 1,648 and 1,710 cm^–1^ and assigned to the to the lignin substructures such as coniferyl alcohol and coniferyl aldehyde. The peak heights and areas were normalized over the aromatic ring stretching peak height at 1,601 cm^–1^ to compensate for the differences in band intensity due to changes in focal plane. Before normalization, to discard outliers given by negative values and values close to zero, peak areas lower than 1 and peak heights lower than 0.01 were set to 1. Only a small fraction of the areas and heights were rejected as outliers by means of this sorting method (<1%). For the spectra assigned to the CCML cluster, a threshold clustering was applied to further distinguish between the cell corners (CC) and the S1–S3 layers and the middle lamella (S1S3ML). The spectra with ratio 2,898 cm^–1^/1,601 cm^–1^ higher than *x* where assigned to S1S3ML, the rest to the CC cluster. *x* was individually set for each image after visual inspection, and varied between 1.8 and 2.

Due to the high content of noise, a statistical test was done to exclude unreliable information from the main peaks of interest, identified as the C=O and O-D stretching vibrations. The total raw sum of C=O (1,710–1,780 cm^–1^) and O-D (2,300–2,685 cm^–1^) counts were linearly baseline corrected. By evaluating the baselined raw sum of the peaks over the noise of the spectrum, each image pixel was labeled as significant or not, regarding the O-D and CO signals. The non-significant pixels contributed as null values in the averages. The pixels assigned to a mere fluctuation of noise were the ones in which the following expression was not fulfilled:


(2) $$\frac{p_{\text{raw,events}}}{\sigma_{\text{noise}}}>3.5$$

with *p*_*raw,events*_ the total sum of the counts (raw spectra, linearly baselined) and considering the background to be 0±σ_noise_events. The σ_noise_ was computed as the standard deviation of the difference between the raw and the reduced signal (PCA, first 3 components). Considering the Poisson statistic of the event of Raman Stokes scattering from a functional group, the 3.5σ_noise_ threshold is a cautious one (Barlow, 1993). A null value was assigned to the spectra belonging to the pixels of lumina of tracheids and ray cells, as well as to the pixels in which the peak of interest was not significantly greater than the background noise.

## Results and discussion

### General observations about acetylation, hydroxyl groups and moisture

The average spectra for the acetylated samples showed higher intensity than the one for untreated wood at approximately 645, 910, 1,735, and 2,941 cm^–1^, as previously reported by Digaitis et al. (2021) for acetylated spruce wood cell walls (Figure 1). These peaks were, respectively, assigned to O-C=O in plane deformation, H-C=C and H-C=O bending, C=O carbonyl stretching vibration and C-H stretching vibration, which are related to acetylation (Wiley and Atalla, 1987; Adebajo et al., 2006; Bock and Gierlinger, 2019). As expected, these peaks did not vary significantly over the three hygroscopic states of the same types of wood. Among those, the peak area assigned to the C=O carbonyl stretching vibration, calculated between 1,710 and 1,780 cm^–1^ (yellow band in Figure 1), was used as peak of interest to characterize acetylation (Adebajo et al., 2006). The C=O peak intensity is visibly the highest in uniformly acetylated wood (highest degree of wood cell wall acetylation, *R*_mod_ = 0.142 g/g), while the interface acetylated had the second highest peak intensity (lower degree of wood cell wall acetylation, *R*_mod_ = 0.113 g/g), in all the three hygroscopic states. This is because the interface acetylation only acts at the lumen-cell wall interface, i.e., the somewhat lower peak height compared to uniformly acetylated wood is a dilution effect of the spectral averaging.

The O-D signal (blue shading in Figure 1) is from the deuterated hydroxyl groups and moisture within the cell walls. Since each D_2_O water molecule contains two O-D functionalities that contributes to the Raman signal, the measured O-D signal reflects the accessible hydroxyls plus two times the concentration of water molecules. Please refer to Supplementary Material, Section 3 for an extended discussion of this point. In the average spectra, the O-D signal was seen at 5% RH and it increased for higher RH levels for all the types of wood studied. These observations indicate that successful deuteration and moisture uptake in the cell walls was achieved.

The O-H band (green shading in Figure 1) derives from the un-deuterated hydroxyl groups. Since the wood specimens were exposed to both liquid D_2_O and high D_2_O vapor pressure for prolonged time, it can be assumed that all water-accessible hydroxyl groups were deuterated. Consequently, the O-H signal relates to the hydroxyls inaccessible to water, and these groups are mainly found inside the cellulose microfibrils (Hofstetter et al., 2006; Salmén and Bergström, 2009). No outstanding variations can be pointed out from this band, neither between the different types of wood nor between the hygroscopic states, except for a slightly higher O-H peak at 5% RH for the untreated samples.

### Distribution of acetyl groups and deuterium within cell walls

**FIGURE 2:**
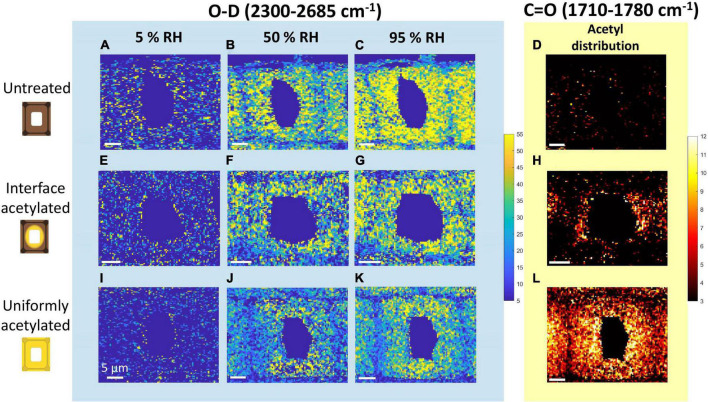
Intensity maps of the Raman peaks assigned to moisture and acetylation of untreated **(A–D)**, interface acetylated **(E–H)** and uniformly acetylated wood **(I–L)**. The three columns of maps on the left show O-D stretching (2,300–2,685 cm^–1^) intensity scanned at 5% (first column), 50% (second column), and 95% RH (third column). The column on the right shows intensity maps of C=O stretching (1,710–1,780 cm^–1^). For further clarity the backgrounds of the O-D and C=O intensity maps are color-coded as the band in Figure 1, i.e., respectively, blue and yellow. All the maps of O-D share the same intensity scale, as do all maps of C=O.

Figure 2 shows the intensity maps of the peak areas assigned to O-D (Figures 2A–C,E–G,I–K) and C=O stretching (Figures 2D,H,L) for one tracheid from each treatment. Due to the complex composition of wood and the lateral resolution of Raman micro-spectroscopy, the spectra from wood specimens often contain overlapping information. However, for this study the intensity maps of O-D and C=O vibrations could with high certainty be related to deuterated hydroxyls plus moisture, and acetyl esters, respectively.

In interface acetylated wood (Figure 2H), the C=O distribution was mainly concentrated in the cell wall area around the lumen, while it was basically absent in untreated wood (Figure 2D) and uniformly distributed in the uniformly acetylated wood (Figure 2L). These maps also indicate that the maximum C=O signal is of the same magnitude for the two different types of acetylation. As also seen from the average spectra in Figure 1, the intensity maps of C=O confirm that intensity and distribution between different hygroscopic states of the same tracheid does not vary significantly, as no acetyl esters are introduced or washed out during the moisture conditioning of the samples.

All types of wood materials showed an increase in the O-D signal over the whole cell wall, when going from nearly dry (5% RH) to more moist hygroscopic states. Since the amount of water-accessible hydroxyl groups does not vary over the three hygroscopic states (Altgen and Rautkari, 2021), the difference between the images of the same material were solely due to the moisture uptake. The major difference between modified and untreated wood was in the intensity and distribution of the O-D signal. The untreated wood (Figures 2A–C) had higher and more even distribution of the O-D signal over the secondary cell wall at every hygroscopic state than what was seen for the interface acetylated (Figures 2E–G) and uniformly acetylated wood (Figures 2I–K). Furthermore, the two types of acetylated wood showed a more uneven distribution of the O-D signal in the cell wall than the untreated wood, and lower values of O-D seemed to be associated with higher values of C=O signal intensity, i.e., the degree of acetylation (Figure 2F with Figure 2H and Supplementary Figure 4). Overall, the O-D intensity maps showed that the moisture uptake was reduced in the acetylated samples, illustrating the usefulness of the experimental setup.

### Quantification of acetyl esters and moisture present within individual cell wall layers

**FIGURE 3:**
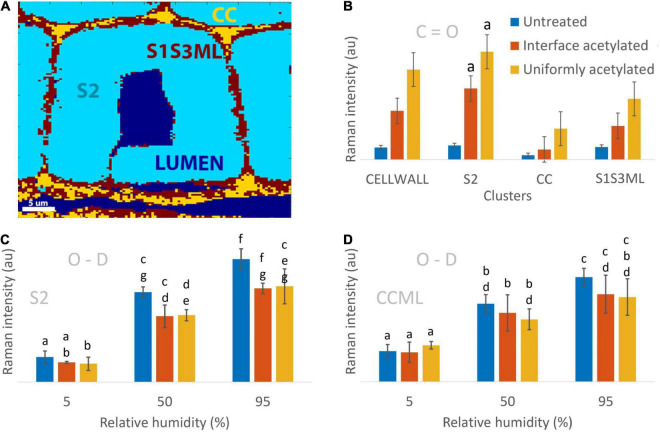
Quantification of the Raman O-D and C=O signals in the different clusters of the cell wall. **(A)** Example of the wood cell clustering depicting LUMEN (tracheid and ray cell lumina), S2 (secondary cell wall), CC (cell corners), S1S3ML (primary and tertiary cell wall, and middle lamella). The CELLWALL cluster (not shown) is the sum of the S2, CC and S1S3ML clusters, i.e., the whole cell wall. **(B)** Average Raman C=O intensity computed for untreated, interface acetylated and uniformly acetylated wood by averaging the images from the three replicates and the three hygroscopic states for each cluster (9 images contributed to the average and standard deviation of each bar). **(C,D)** Average Raman O-D intensity calculated for the three materials in the three hygroscopic states for S2 and CCML clusters. All Raman intensities are normalized over the lignin peak height at 1,601 cm**^–^**^1^. In **(B–D)** untreated samples are illustrated by blue bars, interface acetylated samples by red and uniformly acetylated samples by yellow. The bars include the standard deviation. The bars were compared using a two-tailed pair *t*-test for significance with the null hypothesis of no difference between the two set of data and α = 0.05. The letter *a* on top of the bars in **(B)** indicates that the interface and uniformly acetylated belonging to the S2 cluster were the only results not rejecting the null hypothesis of equal signal for all three groups.

Finally, the clustering analysis that identified and localized different areas of the wood cell walls (Figure 3A) was used to quantify the contribution of moisture and acetylation separately for each of those regions. Figure 3B shows the mean value of the C=O stretching from cell walls of every kind of wood material and specific clusters. In each of the clusters analyzed, the average C=O stretching peak area (associated with acetylation) was highest for the uniformly acetylated samples and lowest for the untreated wood, with the interface acetylation in between, however, without being statistically significant (Figure 3B). To evaluate the reliability of the obtained results, the average Raman C=O signal was compared with the bound acetyl content found in literature. This was done by taking the ratio of the C=O signal of acetylated and untreated wood. For the cell wall cluster, this ratio was 7.4 ± 3.3 for uniformly acetylated and 4.0 ± 1.3 for interface acetylated wood. For comparison, the ratio of bound acetyl concentration in uniformly acetylated and untreated wood is theoretically expected to be 11.9 ± 0.8 and 9.4 ± 0.6 (Supplementary Table 1), which align with experimental data for acetylation of *Radiata pine* (Beck et al., 2017, 2018). Thus, the theoretical ratios were somewhat higher but of the same magnitude as the ratios of the Raman C=O signals.

In the S2 cluster (Figure 3C), the mean O-D values at each of the hygroscopic states of untreated and interface acetylated wood were statistically the same, as well as between interface and uniformly acetylated wood, while results for the untreated wood were different. This is in contrast to the CCML cluster (Figure 3D) where mean O-D values of interface acetylated, uniformly acetylated and untreated wood were all statistically comparable between the same hygroscopic states. Overall, the trends of Figures 3C,D make us speculate that the interface acetylated wood reduces the moisture uptake relatively more in the secondary cell wall than the uniformly acetylated wood, even though the statistics can only partially confirm this claim. The low significance of the data regarding the mean O-D signal suggests that, considering the evidences from the average spectra (Figure 1), the intensity maps (Figure 2) and the trends from the bar charts (Figures 3C,D), three replicas is not enough to overcome the great variability of the material.

To further evaluate the obtained results, the Raman O-D signal at 5% RH was compared with the expected O-D concentration from experimentally determined hydroxyl accessibility and predicted residual moisture. The ratio of the O-D signal of acetylated samples and untreated wood was compared with predicted O-D concentration at 5% RH (Supplementary Table 3). Whereas the latter gave ratios of 0.7 ± 0.1 for interface acetylated and 0.4 ± 0.1 for uniformly acetylated wood, the ratios based on the Raman O-D signal were found to be 0.8 ± 0.3 and 0.9 ± 0.2 for interface and uniformly acetylated wood, respectively. Thus, while the Raman data suggests a decreasing O-D concentration for the acetylated materials, the uniformly acetylated wood exhibited a more intense O-D signal at 5% RH than the predicted values.

The Raman O-D signal from the cell wall cluster (not shown) for each type of material was also compared with the predicted O-D concentration in the different moist states based on experimental data from Digaitis et al. (2021). The values showed the O-D Raman signal and O-D concentration at 50 and 95% RH, normalized over the corresponding values at 5% RH to be of the same order of magnitude (Supplementary Table 2).

Overall, this study demonstrates the effect of acetylation on moisture uptake locally in wood cell walls, and illustrates the possibilities for simultaneously exploring sub-micron level wood chemistry and moisture in wood under hygroscopic conditions.

## Conclusion

A novel experimental set-up was introduced in this study for simultaneous exploration of the sub-micron level cell wall chemistry and moisture in wood under hygroscopic conditions. Analysis of both cell wall-lumen interface acetylated and uniformly acetylated latewood cells of Norway spruce illustrated the local effect of acetyl esters on moisture uptake in different regions of the cell wall, at various levels of relative humidity and with sub-microscale resolution. The results collectively point to the conclusion that moisture is reduced more in highly acetylated areas of the cell wall.

## Data availability statement

The raw data supporting the conclusions of this article will be made available by the authors, without undue reservation.

## Author contributions

AP, ET, RD, LT, MF, and SS: conceptualization. AP, MF, SS, and RD: methodology and material production. AP, LT, and ET: writing—original draft preparation. AP: data acquisition and data analysis. LT, ET, and SS: supervision. All authors have read and agreed to the published version of the manuscript.
