# Supplementary material for: Raman micro-spectroscopy of two types of acetylated Norway spruce wood at controlled relative humidity
Source: Front Plant Sci. 2022 Sep 6;13:986578. doi: 10.3389/fpls.2022.986578 (PMC9486069; doi:10.3389/fpls.2022.986578)
Supplement: Supplementary file 1 [file Data_Sheet_1.pdf]

Supplementary Material

1 Moisturizing system

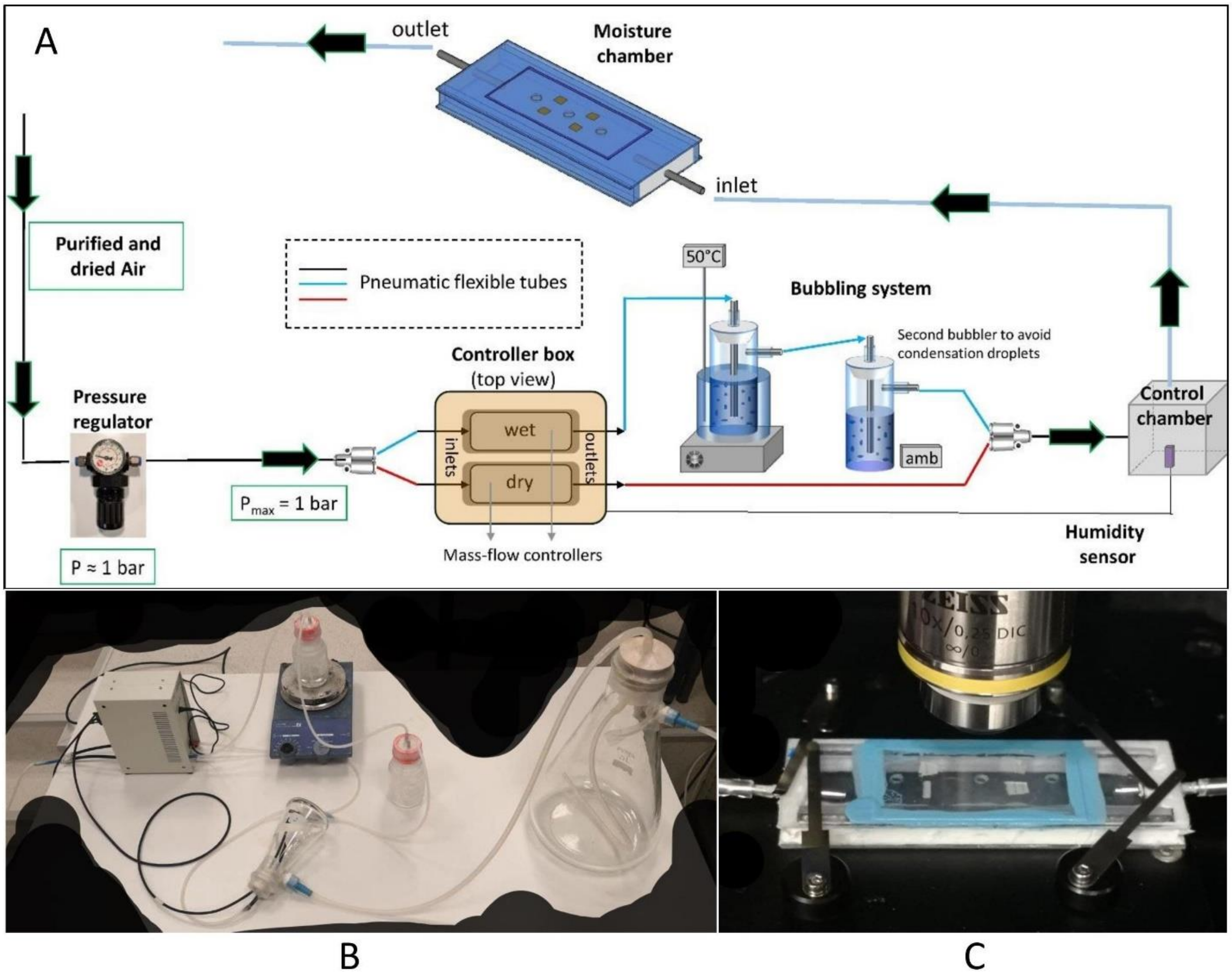

Supplementary Figure 1. A) Schematic illustration of the moisture conditioning system (adapted from SolGelWay, France and reprinted under permission). B) Picture of the controller box, bubbling system and control chamber. The larger flasks (5 L) on the right side of B was introduced to prevent fast oscillations in RH. That flask is missing in the scheme in A. C) Picture of the moisture chamber on the Raman stage, under 10x/0.25 optic.

The wet flux was vapour saturated by letting the flow go through a double-flasks bubbling system containing D_2O (99.98%, Sigma-Aldrich, Munich, Germany). Each bottle has a volume of 250 mL and was equipped with 45 mm solvent delivery caps with a two-ports tubing connector (SIMAX, Czech Republic). The first bottle was placed on a warming plate (RCT basic, IKA labortechnik, Germany) to keep the water at 50 °C and over-saturate the wet flux, while the second was used for condensation purposes and kept at room temperature (20 °C) (Supplementary Figure 1A,B). The dry flux and the wet flux were mixed in a third 250 mL suction bottle (SCHOTT UK ltd, United Kingdom) where an RH sensor (SHT25, Sensirion Ag, Staefa, Switzerland) was placed too. The sensor communicates with the humidity controller box (ACE flow 2.0, SolGelWay, France), which was set to adjust the wet and dry flux to the target relative humidity. Both the sensor and the mixed flux were inserted in the flask through a two-holes rubber lid (Supplementary Figure 1A,B). To

mitigate the fluctuations of the moisture generator, the humid flux was directed from the mixing flux to a 5 L volume suction bottle (SCHOTT UK ltd, United Kingdom) (Supplementary Figure 1A,B), and only then into the moisture chamber (Supplementary Figure 1C,D). The wood cross-sections containing moisture chamber (Supplementary Figure 1C) consists of two interconnecting sub-chambers. The larger sub-chamber is made of two stacked microscopy glass slides kept at a rigid distance by two PMMA sticks (polymethyl methacrylate) in between them with the edges around the glass slides sealed with a silicon glue. Three holes were drilled into the upper side of the larger sub-chamber using a diamond drill (1.5 mm diameter). The smaller, wood cross-sections containing sub-chamber is established by applying a cover glass on top of the larger sub-chamber, including specimens, and sealing the edges with nail polish. The smaller subchamber holding the wood specimens is thus in contact with the larger sub-chamber via the three holes, and the wood cross sections are placed on top of the larger sub-chamber without covering the holes. The larger, bottom moisture sub-chamber has two connections for inlet and outlet of moisture, these are made of 15 mm long stainless-steel pipes with 3 mm and 2 mm of outer and inner diameters. The microscopy glass slides are 80 mm long, 25 mm wide and 1 mm thick. The sticks are 5 mm wide, 70 mm long and 4 mm thick. The overall thickness of the larger bottom sub-chamber is 6 mm and the volume where the humid air flows is 4.2 cm^3. The cover slides are borosilicate glass with 22 mm x 40 mm size and thickness #1 (0.13-0.16 mm, VWR, USA). By assuming that the nail polish reduces the cover glass available area by 1 mm on each side (20 mm x 38 mm) , and a height that ranges between 16 µm and 20 µm (the thickness of the wood specimens), the volume of the wood cross-sections containing sub-chamber is estimated to be between 12-15 mm^3. The moisture chamber was connected to the moisture generator trough silicon tubes of diminishing diameter: from 10 mm (outer diameter, while 8 mm inner diameter) at the outlet of the buffer's flask to 3 mm (outer diameter, while 2 mm of inner diameter) at the inlet of the chamber.

2 Mean spectra of pyridine treaded wood (control)

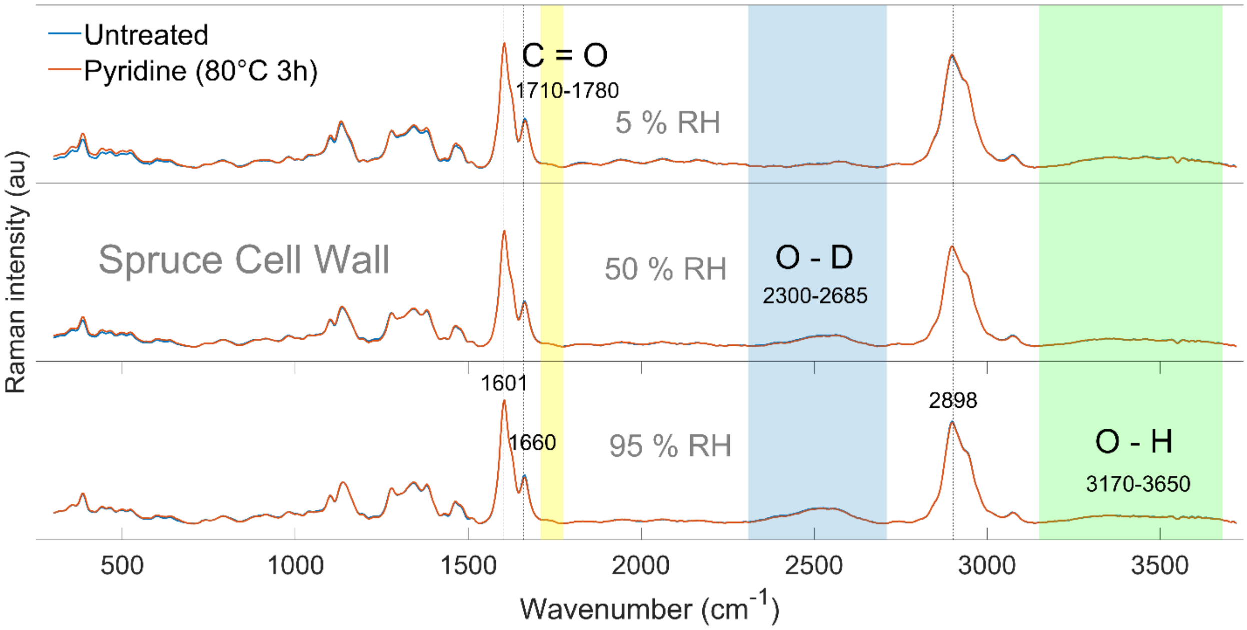

Supplementary Figure 2. Average Raman spectra calculated for untreated (blue) and pyridine treated (red) spruce cell walls (i.e., CELL WALL cluster), conditioned in D_2O vapour at 5% (top), 50% (middle) and 95% RH (bottom). Wavenumber regions of interest assigned to acetylation, moisture and water-inaccessible hydroxyls are shaded respectively in yellow, blue and green. Peaks

of interests are also highlited by dotted lines, and labeled with their exact wavenumber. The spectra of pyridine control samples overlap almost entirely with the spectra of untreated wood throughout the wavenumber range and at all three hygroscopic states.

3 Expected C=O and O-D concentration

To evaluate the reliability of the Raman results, the average values of C=O and O-D signals were compared with the expected values from literature (Beck, Thybring and Thygesen, 2018; Digaitis *et al.*, 2021). The C=O signals of interface and uniformly acetylated wood belonging to CELL WALL, S2 and CCML clusters were compared (Supplementary Table 1) with the acetyl contents of the three types of wood obtained from literature. To permit the comparison, all the values were normalised over the acetyl content or Raman C=O signal of untreated wood. The average C=O signal was normalised with the average C=O signal of the untreated wood for the respective clusters. The calculated acetyl contents of interface and uniformly acetylated wood were normalised over the acetyl content of untreated wood. The closest estimation of acetyl content we found is from untreated *Radiata pine* wood (Beck, Thybring and Thygesen, 2018), with a value of 0.31 ± 0.02. The acetyl contents of interface and uniformly acetylated wood were obtained by dividing the mass gain from modification by 42.037 g mol^{-1} (molar mass of acetyl group minus the molar mass of hydrogen) and adding the acetyl content of untreated wood. The normalised C=O signal from the cell wall material showed to be in the same order of magnitude as the normalised predicted acetyl content (Supplementary Table 1). Furthermore, the absolute acetyl content and the C=O signal (Supplementary Table 1, green columns) showed good agreement, with minor differences between the acetyl content and the Raman signal for the untreated material. Since the acetyl content of the untreated wood used for normalisation is based on data from another tree species, uncertainty in the theoretical predictions is expected. By looking at the normalised C=O signal belonging to the S2 and CCML clusters, the values of uniformly acetylated and interface acetylated wood are proportionally different. The ratio between normalised C=O from uniformly acetylated wood versus interface acetylated wood (ratio not shown) are higher in the CCML than in S2. The ratios are respectively 2.5 ± 1.6 and 1.5 ± 0.8. This suggests, once again, that the uniformly acetylated wood is relatively more acetylated in the CCML than the interface acetylated wood.

Supplementary Table 1. Comparison between the average values and standard deviation of Raman C=O signal belonging to the CELL WALL, S2 and CCML clusters with the values of acetyl content of untreated wood (Beck, Thybring and Thygesen, 2018) and of acetylated wood. The error range indicates the standard deviation.

		Acetyl content from literature (Beck, Thybring and Thygesen, 2018)		***Raman C=O signal (this study)***					
				CELL WALL		***S2***		***CCML***	
Material	***Mass gain***	***Acetyl Content***	***Normalized Acetyl Content***	***C=O signal***	***Normalized C=O signal***	***C=O signal***	***Normalized C=O signal***	***C=O signal***	***Normalized C=O signal***
	(g/g)	(mmol/g)	(-)	(au)	(-)	(au)	(-)	(au)	(-)
**Untreated**	0.00	0.3 (Beck *et al.*, 2018)	1.0 ± 0.0	0.5 ± 0.1	1.0 ± 0.0	0.6 ± 0.1	1.0 ± 0.0	0.4 ± 0.1	1.0 ± 0.0
**Interface**	0.11	2.9	9.4 ± 0.6	2.0 ± 0.5	4.0 ± 1.3	2.9 ± 0.7	5.0 ± 1.7	0.8 ± 0.2	2.3 ± 0.9
**Uniform**	0.14	3.7	11.9 ± 0.8	3.7 ± 1.4	7.4 ± 3.3	4.5 ± 1.3	7.6 ± 2.9	2.1 ± 0.8	5.8 ± 2.8

The Raman O-D signal belonging to the cell wall was compared with the O-D concentration calculated form the values available in literature (Digaitis *et al.*, 2021). The values at 50% and 95% RH were normalised with the O-D concentration at 5% RH (Supplementary Table 2). The

normalised Raman O-D signal is of the same order of magnitude as the normalised predicted O-D concentration.

The normalised O-D concentration at each hygroscopic level was calculated by the sum of the expected residual moisture (g/g) at that hygroscopic state and the hydroxyl accessibility (mmol/g). Since a D_2O molecule contains two O-D functionalities, the converted residual moisture (mmol/g) was doubled and summed to the hydroxyl accessibility. The hydroxyl accessibility and the residual moisture content at 50% and 95% RH for the materials studied is based on data by (Digaitis *et al.*, 2021). The moisture content at 5% RH was calculated by fitting respectively a line and the ABC isotherm (Zelinka, Glass and Thybring, 2018) to the desorption isotherm data from (Digaitis *et al.*, 2021).

From Supplementary Table 3 it is possible to get an idea of the O-D concentration from the residual moisture that contributes to the signal at 5% RH. The calculated O-D concentration of interface and uniformly acetylated wood at 5% RH were normalised over the untreated wood and compared to the corresponding Raman O-D signal. At 5% RH, the driest state recorded in this study, the moisture accounts for roughly 30% of the O-D concentration. The normalised Raman O-D signal at 5% RH of uniformly acetylated wood is roughly two times the expected normalised O-D concentration at 5% RH of the same material (Supplementary Table 3). However, the analogous values from interface acetylated wood showed good agreement with respectively 0.7 ± 0.1 and 0.8 ± 0.3 for the normalised predicted O-D concentration and the normalised Raman O-D signal from the cell wall material.

Supplementary Table 2. Comparison of predicted O-D concentration with the Raman O-D signal of the cell wall. Values of hydroxyl accessibility (Digaitis *et al.*, 2021) determined for the various materials as well as predictions of the residual moisture content for the three materials in the different moisture states is given in the table. The Raman signal and predicted O-D concentration were normalised across the different moisture states with the value at 5% RH. The error range indicates the standard deviation.

		Residual moisture and hydroxyl accessibility (Digaitis *et al.*, 2021)				***Predicted O-D concentration***			***Normalised predicted O-D concentration***			***Normalised Raman O-D signal CELLWALL***		
Material	***Mass gain*** (g/g)	***Hydroxyl accessibility*** (mmol/g)	***5% RH*** (mmol/g)	***50% RH*** (mmol/g)	***95% RH*** (mmol/g)	***5% RH*** (mmol/g)	***50% RH*** (mmol/g)	***95% RH*** (mmol/g)	***5% RH*** (-)	***50% RH*** (-)	***95% RH*** (-)	***5% RH*** (-)	***50% RH*** (-)	***95% RH*** (-)
**Untreated**	0.00	9.5 ± 0.1	2.1 ± 0.6	11	28	13.6 ± 1.3	31.5 ± 0.1	65.5 ± 0.1	1	2.3 ± 0.2	4.8 ± 0.5	1	3.4 ± 0.7	4.6 ± 0.6
**Interface**	0.11	6.4 ± 0.4	1.3 ± 0.2	8.5	22.4	9 ± 0.8	23.4 ± 0.4	51.2 ± 0.4	1	2.6 ± 0.2	5.7 ± 0.5	1	2.9 ± 0.2	4.2 ± 0.2
**Uniform**	0.14	4.2 ± 0.7	0.8 ± 0.1	6.3	16.4	5.8 ± 0.8	16.8 ± 0.7	37 ± 0.7	1	2.9 ± 0.2	6.4 ± 0.9	1	3 ± 0.5	4.2 ± 0.6

Supplementary Table 3. Comparison of the predicted O-D concentration with the Raman O-D signal at 5% RH across the different materials. Values of predicted O-D concentrations are similar to those in Supplementary Table 2, but the values are normalised with the O-D concentration in the untreated material. The error range indicates the standard deviation.

		Residual moisture at 5% RH and hydroxyl accessibility (Digaitis et al., 2021)				***Raman O-D signal (this study), CELL WALL cluster***	
Material	***Mass gain*** (g/g)	***Hydroxyl accessibility*** (mmol/g)	***Residual moisture*** (mmol/g)	***Predicted O-D concentration*** (mmol/g)	***Normalised predicted O-D concentration*** (-)	***Absolute signal*** (au)	***Normalised signal*** (-)
**Untreated**	0.00	9.5 ± 0.1	2.1 ± 0.6	13.6 ± 1.3	1.0 ± 0.0	7.9 ± 2.1	1.0 ± 0.0
**Interface**	0.11	6.4 ± 0.4	1.3 ± 0.2	9.0 ± 0.8	0.7 ± 0.1	6.6 ± 1.0	0.8 ± 0.3
**Uniform**	0.14	4.2 ± 0.7	0.8 ± 0.1	5.8 ± 0.8	0.4 ± 0.1	7.0 ± 1.4	0.9 ± 0.2

4 O-H and lignin intensity maps

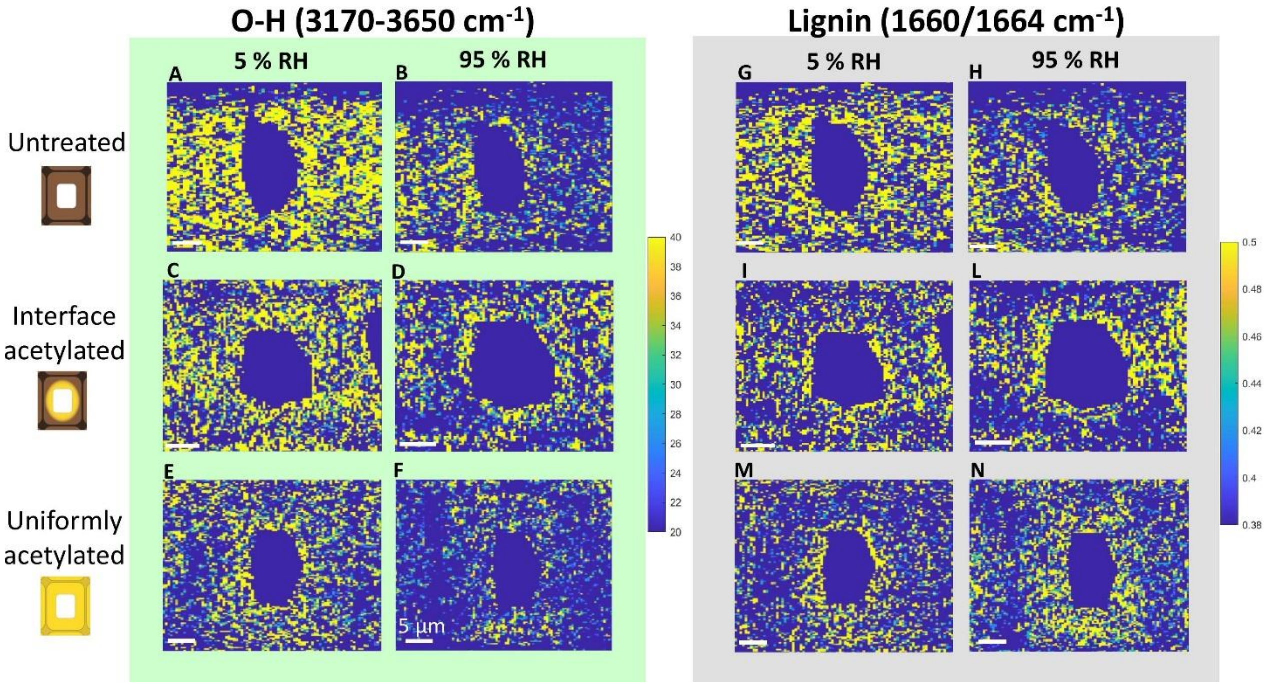

Supplementary Figure 3. Intensity maps of the Raman peak assigned to water-inaccessible hydroxyls and lignin of untreated (blue frame A,B,G,H), interface acetylated (red frame C,D,I,L) and uniformly acetylated wood (yellow frame E,F,M,N). A-F) Maps of O-H stretching (3170-3650 cm^{-1}) scanned at 5% (A,C,E) and 95% RH (B,D,F). G-N) Maps of 1660-1664 cm^{-1} scanned at 5% (G,I,M) and 95% RH (H,L,N). For further clarity the background of the O-H and lignin intensity maps is colour-coded as the band or line in Figure 1, green and gray respectively. All maps of O-H share the same colour-coded intensity scale, and similar for the maps of lignin.

In Supplementary Figure 3 we report the intensity maps of the O-H peak area and lignin peak height (1660-1664 cm^{-1}) at 5% and 95% RH. They have been generated with the intent to localise the small differences in intensity spotted in Figure 1. It is to bear in mind that all the Raman signal in consideration have been normalised over the lignin peak at 1601 cm^{-1}, to correct from differences in intensity due to changes in focal plane.

Looking at the difference between the two hygroscopic states at 5% and 95% RH the O-H signal is reduced in intensity in all types of wood (Supplementary Figure 3A-F). In the acetylated samples the effect is less visible, even if it is still present (Supplementary Figure 3C-F). One possibility for the reduced intensity of the O-H signal at 5% compared to 95% RH is swelling of the cell wall structure, which increases the space between the cellulose microfibrils. In a study from (Papadopoulos, Hill and Gkaraveli, 2004) the volumetric swelling between dry and moisture conditioned wood at 93% RH of untreated and acetylated Corsican pine (R_{mod} = 0.16) was found to be 12% and 7% respectively, where the swelling of the acetylated samples included only the swelling due to moisture uptake.

The cell wall areas where the acetylation is higher (see Figure 2 H, L), show lower reduction in O-H intensity when going from 5% (Supplementary Figure 2 C, E) to 95% RH (Supplementary Figure 2 D, F), suggesting a minor swelling of those regions of the cell wall.

The swelling in the cell wall chemistry was further explored by the intensity maps of the lignin peak at 1660-1664 cm^{-1} (Supplementary Figure 3 G-N). All the samples showed a dilution from the 5% RH intensity maps to the 95% RH maps. For the untreated wood the reduction is similar to the one seen for the O-H signal, while the acetylated samples (in particular, the acetylated areas of the acetylated samples) showed more a rearrangements-like changing of the intensity in the lignin maps between 5% and 95% RH (Supplementary Figure 2 I-N). Addressing the reason of these differences in dilution between OH and lignin intensity maps is a difficult exercise though, as the picture around the behaviour of microfibrils and lignin are still under discussion. Both lignin and cellulose microfibrils gets completely surrounded by moisture, especially in the secondary cell wall, as immersed in a very hydrophilic environment (Terrett *et al.*, 2019; Kirui *et al.*, 2022). At increasing moisture content lignin is thought to increase its volume while the cross section of softwood cellulose microfibrils shrinks (Rafsanjani *et al.*, 2014; Kulasinski *et al.*, 2015; Salmén *et al.*, 2021; Ibach, Plaza and Pingali, 2022; Salmén, 2022). However, the measured strain in microfibrillar lateral lattice strains is measured to be of 0.6% between a variation from 0% and 100% RH (Zabler *et al.*, 2010; Salmén *et al.*, 2021), which is a too small change to be seen by Raman micro-spectroscopy. We can therefore limit the discussion about O-H and lignin (1660-1664) swelling only to the evidence of the reduced dilution of the latter at increasing moisture contents (Supplementary Figure 3), without though addressing the main phenomena behind it.

5 Determining the time to achieve equilibrium moisture state

To verify how long the sample took to reach a dimensional stability, i.e. stops swelling, we performed a preliminary test where the specimens were exposed to humid air inside the moisture chamber. The test consisted in taking screenshots (several screenshots taken over 7 hours using a widefield microscope, 50x air immersion objective) to track the swelling of a dry single cell wall when exposed to humid air (95% RH). By tracking the movement of a single cell wall most of the swelling was observed in the first 40 minutes of conditioning (85% to 95% of the swelling happened in the first 40 minutes, respectively in the first and the second replica) and the swelling stopped after 6 hours of conditioning. The experiment was performed on two cross section of Norway spruce sapwood (*Picea abies* (L.) Karst), microtome cut (RM2255, Leica Biosystems, Wetzlar, Germany) with a thickness of 20µm, and using H_2O (MilliQ water) both for the preparation and the conditioning. The movement of the cell wall was associated with the water-induced swelling and the absorption of water molecules by the tracheid's cell walls. Therefore, we assumed that the moisture equilibrium could be reached after 6 hours and we chose a 'safe' conditioning time of 12 h, twice the time after which the micro swelling was imperceptible by our microscope.

Furthermore, it is worth to bear in mind that during the actual measurements with D_2O vapour, four specimens were sitting in the moisture chamber during each run, and were scanned one after the other, so the first one was scanned after 12 h of conditioning while, the next one after 12 h plus the time to scan the first one, which was on average 30 to 40 minutes, and so on. As we could no longer observe swelling after 6 h, and started all imaging after at least 12 h, we believe that the difference in conditioning time between the first and the last specimen imaged did not affect the moisture content to an extent that could be recorded by our measurements.

6 Additional figures showing the local effect of C = O on O – D

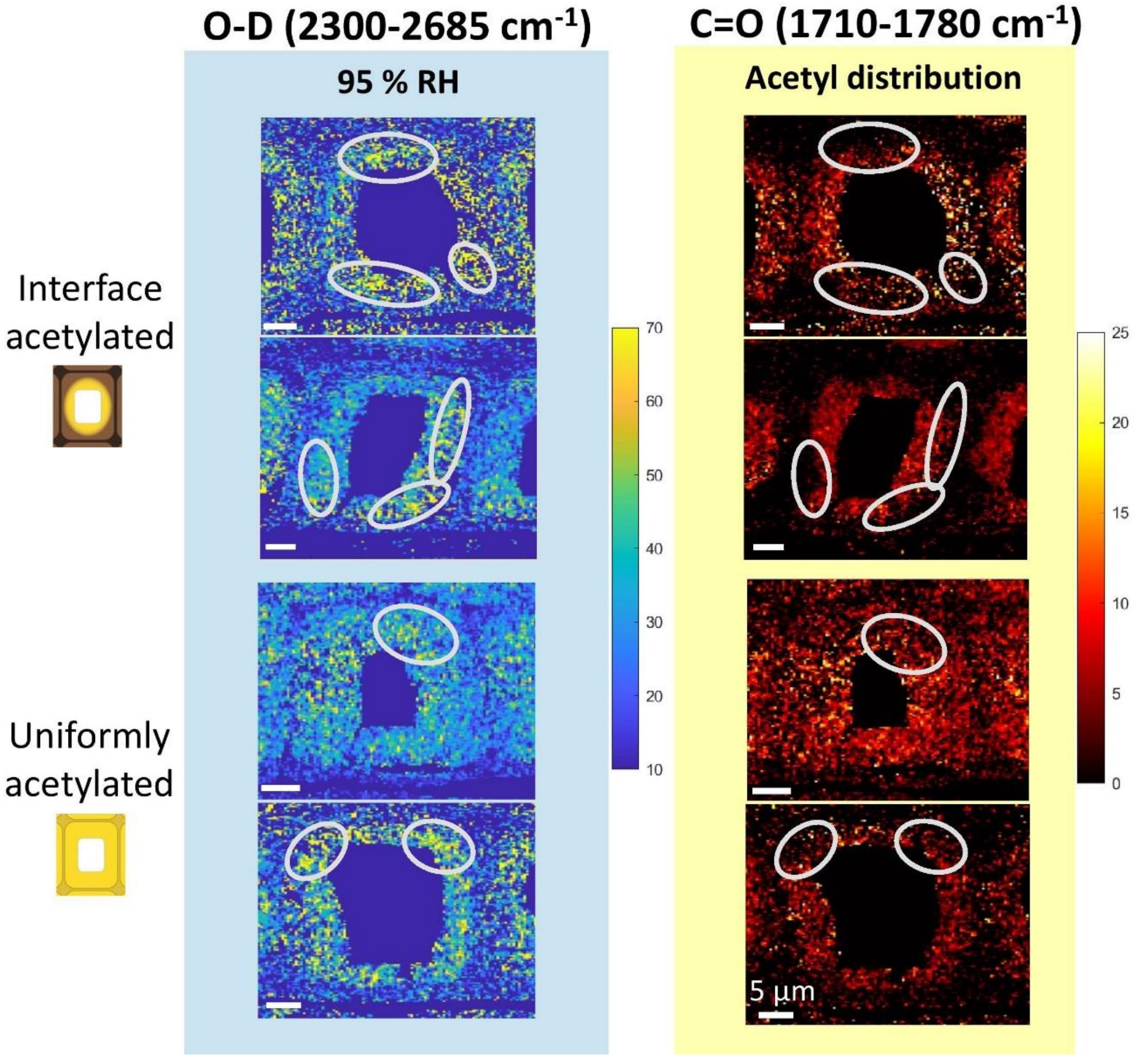

Supplementary Figure 4. Intensity maps of the Raman peaks assigned to moisture and acetylation of interface acetylated and uniformly acetylated wood. The left column of maps show O-D stretching (2300-2685 cm^{-1}) intensity scanned at 95% RH. The column on the right shows intensity maps of C=O stretching (1710-1780 cm^{-1}). For further clarity the backgrounds of the O-D and C=O intensity maps are colour-coded as the band in Figure 1, i.e. respectively blue and yellow. All the maps of O-D share the same intensity scale, as do all maps of C=O. Helliptical shapes highlight the regions of the cell wall where the association of higher values of O-D signal with lower values C = O intensity are most visible.

7 Additional general observations about lignin and CH

Additional observation can be done regarding the average spectra in Figure 1. For example, the lignin peak at 1660 cm^{-1} assigned to the C=O and C=C stretching of lignin substructures is red-shifted 2-4 wavenumbers in the acetylated samples compared to the unmodified wood. The intensity of the peaks decreases at higher moisture contents, with more pronounced decrease in the unmodified wood between 5% and 50% RH than in the acetylated samples. This "dilution" is analogous to the C-H peak intensity at 2898-2902 cm^{-1}, where the unmodified samples show more pronounced decrease than acetylated samples, especially between 5% and 50% RH.

8 Bibliography

Adobes-Vidal, M., Frey, M. and Keplinger, T. (2020) 'Atomic force microscopy imaging of delignified secondary cell walls in liquid conditions facilitates interpretation of wood ultrastructure', *Journal of Structural Biology*. Elsevier, 211(2), p. 107532. doi: 10.1016/j.jsb.2020.107532.

Beck, G. *et al.* (2018) 'Characterization of moisture in acetylated and propionylated radiata pine using low-field nuclear magnetic resonance (LFNMR) relaxometry', *Holzforschung*, 72(3), pp. 225–233. doi: 10.1515/hf-2017-0072.

Beck, G., Thybring, E. E. and Thygesen, L. G. (2018) 'Brown-rot fungal degradation and de-acetylation of acetylated wood', *International Biodeterioration and Biodegradation*. Elsevier Ltd, 135(1431), pp. 62–70. doi: 10.1016/j.ibiod.2018.09.009.

Berglund, J. *et al.* (2020) 'Wood hemicelluloses exert distinct biomechanical contributions to cellulose fibrillar networks', *Nature Communications*. Springer US, 11(1), pp. 1–16. doi: 10.1038/s41467-020-18390-z.

Digaitis, R. *et al.* (2021) 'Targeted acetylation of wood: a tool for tuning wood-water interactions', *Cellulose*, 28(12), pp. 8009–8025. doi: 10.1007/s10570-021-04033-z.

Du, X. *et al.* (2014) 'Analysis of lignin–carbohydrate and lignin–lignin linkages after hydrolase treatment of xylan–lignin, glucomannan–lignin and glucan–lignin complexes from spruce wood', *Planta*, 239(5), pp. 1079–1090. doi: 10.1007/s00425-014-2037-y.

Ibach, R. E., Plaza, N. Z. and Pingali, S. V. (2022) 'Small Angle Neutron Scattering Reveals Wood Nanostructural Features in Decay Resistant Chemically Modified Wood', *Frontiers in Forests and Global Change*, 4(January), pp. 1–11. doi: 10.3389/ffgc.2021.814086.

Kirui, A. *et al.* (2022) 'Carbohydrate-aromatic interface and molecular architecture of lignocellulose', *Nature Communications*. Springer US, 13(1), pp. 1–12. doi: 10.1038/s41467-022-28165-3.

Kulasinski, K. *et al.* (2015) 'Water Adsorption in Wood Microfibril-Hemicellulose System: Role of the Crystalline-Amorphous Interface', *Biomacromolecules*, 16(9), pp. 2972–2978. doi: 10.1021/acs.biomac.5b00878.

Papadopoulos, A. N., Hill, C. A. S. and Gkaraveli, A. (2004) 'Analysis of the swelling behaviour of chemically modified softwood: A novel approach', *Holz als Roh - und Werkstoff*, 62(2), pp. 107–112. doi: 10.1007/s00107-003-0448-8.

Rafsanjani, A. *et al.* (2014) 'Hygroscopic swelling and shrinkage of latewood cell wall micropillars reveal ultrastructural anisotropy', *Journal of the Royal Society Interface*, 11(95). doi: 10.1098/rsif.2014.0126.

Salmén, L. *et al.* (2021) 'Moisture induced straining of the cellulosic microfibril', *Cellulose*, 28(6), pp. 3347–3357. doi: 10.1007/s10570-021-03712-1.

Salmén, L. (2022) 'On the organization of hemicelluloses in the wood cell wall', *Cellulose*, 29(3), pp. 1349–1355. doi: 10.1007/s10570-022-04425-9.

Terrett, O. M. *et al.* (2019) 'Molecular architecture of softwood revealed by solid-state NMR',

Nature Communications, 10(1), pp. 1–11. doi: 10.1038/s41467-019-12979-9.

Zabler, S. *et al.* (2010) ‘Moisture changes in the plant cell wall force cellulose crystallites to deform’, *Journal of Structural Biology*. Elsevier Inc., 171(2), pp. 133–141. doi: 10.1016/j.jsb.2010.04.013.

Zelinka, S. L., Glass, S. V. and Thybring, E. E. (2018) ‘Myth versus reality: Do parabolic sorption isotherm models reflect actual wood–water thermodynamics?’, *Wood Science and Technology*. Springer Berlin Heidelberg, 52(6), pp. 1701–1706. doi: 10.1007/s00226-018-1035-9.
